# From marginal croplands to natural habitats: A methodological framework for assessing the restoration potential to enhance wild-bee pollination in agricultural landscapes

**DOI:** 10.1007/s10980-024-01993-y

**Published:** 2024-11-12

**Authors:** Gabriela María Torchio, Jérôme Cimon-Morin, Poliana Mendes, Jean-Olivier Goyette, Amanda M. Schwantes, Miguel Arias-Patino, Elena M. Bennett, Catherine Destrempes, Stéphanie Pellerin, Monique Poulin

**Affiliations:** 1 Département de Phytologie, Faculté des Sciences de L’Agriculture et de L’Alimentation, Université Laval, 2425 Rue de L’Agriculture, Québec, QC G1V 0A6 Canada; 2 Quebec Centre for Biodiversity Science, McGill University, 1205 Dr. Penfield Avenue, Montreal, QC H3A 1B1 Canada; 3 Centre d’Étude de la Forêt, Université du Québec À Montréal, 141 Président-Kennedy, Montréal, QC H2X 1Y4 Canada; 4 Département des Sciences du Bois et de la Forêt, Faculté de Foresterie, de Géographie et de Géomatique, Université Laval, 2405 Rue de La Terrasse, Québec, QC G1V 0A6 Canada; 5 Département des Sciences Naturelles et Institut des Sciences de la Forêt Tempérée (ISFORT), Université du Québec en Outaouais (UQO), 58 Rue Principale, Ripon, QC J0V 1V0 Canada; 6 Department of Ecology and Evolutionary Biology, University of Toronto, 25 Willcocks Street, Toronto, ON M5S 3B2 Canada; 7 Natural Resources and Environmental Studies Institute, University of Northern British Columbia, 3333 University Way, Prince George, BC V2N 4Z9 Canada; 8 Department of Natural Resource Sciences, McGill University, 21111 Lakeshore Road, Sainte-Anne-de-Bellevue, QC H9X 3V9 Canada; 9 McGill School of Environment, McGill University, 21111 Lakeshore Road, Sainte-Anne-de-Bellevue, QC H9X 3V9 Canada; 10 Institut de Recherche en Biologie Végétale, Université de Montréal and Jardin Botanique de Montréal, 4101 Rue Sherbrooke Est, Montréal, QC H1X 2B2 Canada

**Keywords:** Pollination, Ecosystem services, Agriculture, Restoration, Scenarios

## Abstract

**Context:**

Intensive agriculture increases crop yields, but harms biodiversity and associated ecosystem services such as pollination. To sustain wild-bee pollination in intensive agricultural landscapes, a minimum of (semi-) natural habitat is needed in the vicinity of crop fields. However, restoration of (semi-) natural habitat is a challenge, especially when most land is allocated to commodity production.

**Objectives:**

To evaluate the restoration potential of marginal lands to enhance pollination in intensive agricultural landscapes.

**Methods:**

We simulated restoration scenarios in marginal agricultural lands (abandoned and degraded fields, and field edges) in La Vallée-du-Richelieu (Quebec, Canada), aimed at enhancing pollination provision and increasing (semi-) natural habitat coverage by at least 20% within 1 km from crop fields, the estimated minimum amount required to sustain wild-bee populations. We then evaluated the extent to which restoration targets were reached in our scenarios.

**Results:**

More than half of the agricultural region studied remained with less than 20% (semi-) natural area coverage, and wild-bee pollination provision could not be ensured across the whole agricultural region after restoration. However, our results show that there is still an important potential for increasing natural habitat coverage by restoring marginal agricultural lands alone.

**Conclusion:**

Restoration of marginal lands has a key role to play in the transition towards multifunctionality of production landscapes but might not be sufficient to achieve goals such as those adopted at the COP15 (e.g., restoring 30% of degraded land). Our framework can assist landscape planners in evaluating the restoration potential of agricultural landscapes, as well as its limitations.

**Supplementary Information:**

The online version contains supplementary material available at 10.1007/s10980-024-01993-y.

## Introduction

Since the Green Revolution, agricultural intensification has allowed for large increases in crop yields (Tilman et al. 2002). Intensive agriculture practices typically include monocultures, increased mechanization, and heavy use of pesticides and fertilizers (Ramankutty et al. 2018; Bennett et al. 2021). This has resulted in agricultural landscapes that are homogeneous, have few natural areas, low biodiversity and provide less non-crop ecosystem services (ES) (Foley et al. 2005; Tscharntke et al. 2005; Power 2010). In this context, there is a need for plans to make agricultural landscapes more multifunctional–capable of simultaneously sustaining biodiversity and providing food, materials, and other ecosystem services such as recreation or carbon storage (Lovell and Johnston 2009; Cimon-Morin et al. 2021; Garibaldi et al. 2023).

The worldwide decline in wild-bee pollinators in agricultural landscapes is of significant concern (Potts et al. 2010a). Pollinators’ disappearance forecasts a scenario of deficits in crop production and diversity, given that around 75% of food crops rely on insect pollination to varying degrees, with the majority of this service provided by wild bees (Klein et al. 2007 but see Reilly et al. 2024). There are many drivers of bee decline in agricultural landscapes, including the loss of (semi-) natural habitats (Steffan-Dewenter et al. 2002; Kremen et al. 2002), increased use of pesticides (Nicholson et al. 2023), loss of feeding resources due to weed reduction (IPBES 2016), disease outbreaks in managed hives that can spread to wild pollinators (Fürst et al. 2014), and impacts of climate change (Potts et al. 2010a; Wilson Rankin et al. 2020). Managed (domesticated) bee colonies, which have been used to compensate for pollination deficits, have also experienced significant collapses, mainly due to pathogen spread, particularly in Europe and North America (Potts et al. 2010b). Consequently, addressing the root causes of bee decline is crucial to ensure provision of pollination in the future (Garibaldi et al. 2014; Pywell et al. 2015).

Wild-bee pollination can be enhanced by increasing (semi-) natural habitat (henceforth, *natural habitat* only) within the agricultural landscape (Morandin and Kremen 2013; Raderschall et al. 2021). It has been shown empirically that pollination indicators such as bee richness and abundance, flower visitation rate, and fruit and seed set usually decrease with distance from natural areas (Carvalheiro et al. 2010; Garibaldi et al. 2011; Martins et al. 2015), and are positively correlated with the proportion of natural habitat (Raderschall et al. 2021; Shi et al. 2021). Wild-bee pollination provision occurs when the pollination supply (the capacity of the landscape to supply bee pollinators) meets the demand for it (the need for pollination of some crops by bees), and for which it is mandatory that pollinators are capable of reaching and effectively pollinating crops (Metzger et al. 2021). It follows that wild-bee pollination provision depends on at least two main conditions to be met in the landscape: First, nesting habitat (e.g., woody vegetation for cavity nesters, or less managed soil for ground nesters) must be located in proximity of pollination-dependent crops, at distances within the foraging ranges of the pollinating species (i.e., within the flow area of bee pollination). Second, feeding resources such as flowers must be found close enough to fields to attract pollinators and keep them visiting crop fields, although competition and dilution effects are important to consider (Desaegher et al. 2021). Increasing intensity of agricultural production likely reduces availability of both nesting and feeding sites for wild pollinators because of natural habitat degradation (Kremen et al. 2002; Landis 2017). To address this problem, restoration of natural habitat that considers these spatial needs of wild bees can improve the co-occurrence of supply areas (nesting habitat) and demand areas (crops from which bees feed). Ultimately this would lead to enhanced bee abundance and diversity, boosted pollination and increased crop yields (M’Gonigle et al. 2015; Pywell et al. 2015; González-Chaves et al. 2023).

Landscape design principles suggest that patches of natural area should be interspersed within the productive matrix so as to provide enough resources for bees within their foraging and nesting ranges (Brosi et al. 2008; Landis 2017). The amount and configuration of these natural habitats depend on the type of crops considered, field configuration, and the particular traits (e.g. foraging ranges) of the native bee species. For example, organic almond farms in California need at least 10% natural areas within 1 km (Klein et al. 2012) to benefit from wild-bee pollination, while sweet cherry orchards in Belgium reach a maximum of pollination benefits at 15% natural areas within 250 m (Eeraerts 2023). Watermelon needs ≥ 30% of natural areas within 1.2 km to rely exclusively on wild-bee pollination (Kremen et al. 2004). More generally, it has been proposed that a minimum of 20% natural habitat, at scales that depend on the restoration target, support the provision of multiple ES simultaneously (Tscharntke et al. 2012; Garibaldi et al. 2021).

Achieving such broad restoration targets in intensive agricultural landscapes can be challenging, as most land is used for production, and natural habitats may cover as little as 1–5% of the territory (Tscharntke et al. 2005; Klein et al. 2012). In productive agricultural fields, ecological restoration could entail financial losses in the short term, as land is removed from commodity production (Potts et al. 2016; Tittonell et al. 2020, but see Pywell et al. 2015), and there are recovery time lags before benefits from restoration can be perceived (Ortega-Marcos et al. 2022; González-Chaves et al. 2023). Restoration can also increase pressures on other ecosystems if agricultural production is simply displaced elsewhere (Latawiec et al. 2015).

Restoration of abandoned and degraded fields has been proposed as an option to balance agricultural production, biodiversity conservation, and supply of non-crop ES (Rey Benayas et al. 2007; Beyer et al. 2022) while the benefits of managing field edges have also been highlighted (Albrecht et al. 2020; Zamorano et al. 2020; Shi et al. 2021). These lands, which are not dedicated to crop production or where yields are diminishing because of soil degradation, are sometimes referred to as *marginal agricultural fields*, and could be restored to natural habitat. However, the extent to which this approach would contribute to ensuring pollination provision for the agricultural region remains uncertain.

**Fig. 1:**
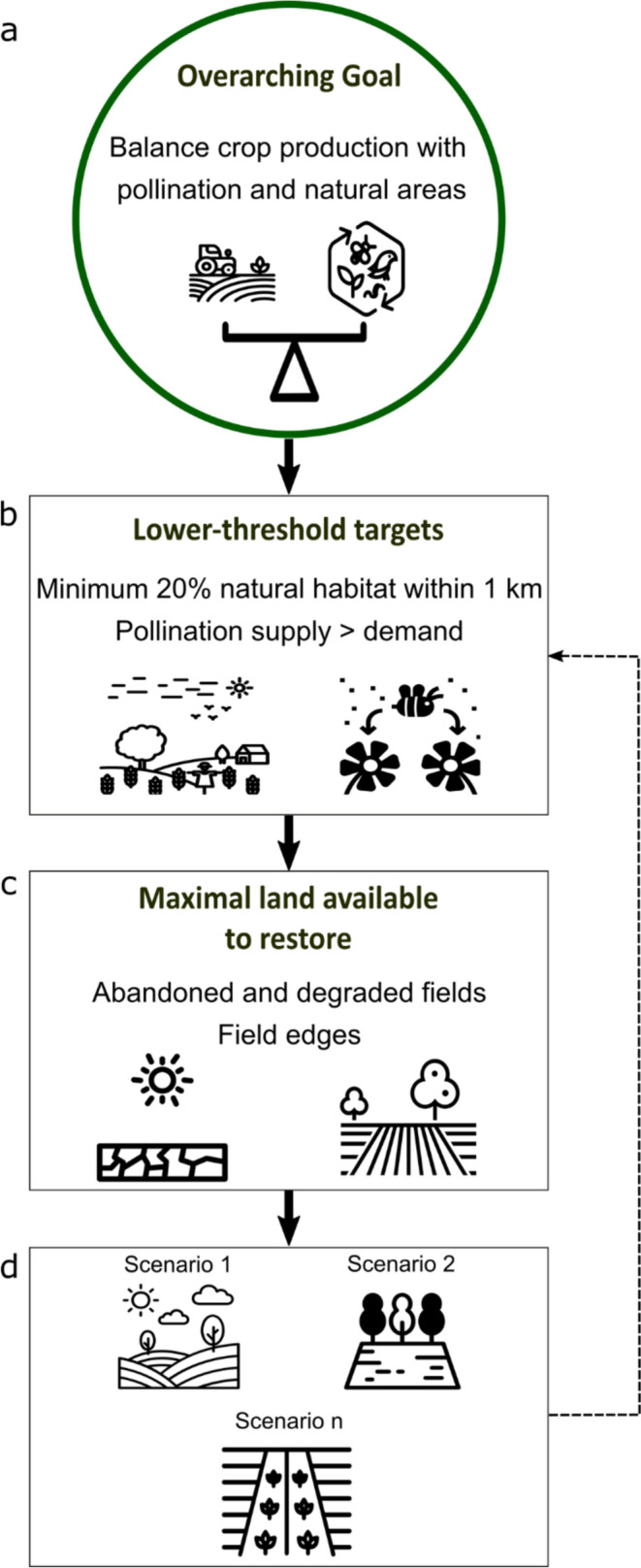
Methodological framework for assessing the restoration potential for enhancing wild-bee pollination within area limitations in agricultural landscapes. In our case study, the overarching goal is to restore natural areas to enhance pollination in agricultural landscapes without taking land out of production (**a**). The *lower-threshold targets* for restoration need to be set. In our case, the lower-threshold targets for restoration were: a minimum of 20% natural area coverage within 1 km and a relative increase in pollination supply over its demand (**b**). The maximum amount of land that can be restored and which land this will be so that restoration does not meaningfully compromise crop production has to be specified (**c**). Restoration scenarios that respect area availability limits are built (for example, restoring only abandoned and degraded fields, restoring field edges alone or combining both approaches), and the extent to which lower-threshold targets were achieved is assessed for each scenario (**d** and dashed arrow). Although the framework is presented as a workflow, steps in boxes (**b**) and (**c**) are more likely to occur in parallel and can be interchanged. Icon sourced from thenounproject.com (License: CC BY 3.0 or Public Domain). Icon creators: Palukx, Ariyanto Deni, Foodicons Collection, Ian Rahmadi Kurniawan, Symbolon, Noura Mbarki, Nithinan Tatah, karyative, Ariyanto Deni, Creative Mahira

We developed a methodological framework to evaluate the potential for enhancing wild-bee pollination in intensive agricultural landscapes (Fig. 1). Within this framework, it is first crucial to identify minimum targets to achieve, which we refer to as *lower-threshold targets.* These could be, for instance, a minimum amount of natural area coverage that is deemed necessary around crop fields to ensure wild-bee pollination. Next, our framework suggests identifying the lands available for restoration and their location. This step is crucial to set the *limits* of restoration, since restoration outcomes–the benefits of restoration–will be constrained by the maximum amount of land available. To illustrate our framework, we applied it to the case study of La Vallée-du-Richelieu (Southern Quebec, Canada), and evaluated whether it is possible to increase wild-bee pollination provision by restoring marginal agricultural lands (abandoned and degraded as well as field edges) alone. We also assessed whether: (1) the establishment of flower strips on field edges complemented a strategy based on restoring only abandoned and degraded fields, and (2) the restoration of all marginal agricultural lands contributed to reaching targets for natural area coverage according to landscape design principles.

## Methods

### Study area

We used the county of La-Vallée-du-Richelieu (LVR) as a case study because it is typical of southeastern Canada, where intensive agriculture is practiced. About 70% of LVR’s territory is cultivated, out of which ~ 76% is used to produce corn (*Zea mays*) and soybean (*Glycine max*) (MRC de la Vallée-du-Richelieu 2022). The rest of the cultivated region is occupied by crops such as vegetables, small fruits including blueberries and strawberries, cereals, as well as orchards, vineyards, and pastures. The mean field size in the region is 6.2 ± 7.1 ha (mean ± SD).

### La Vallée-du-Richelieu’s land use and land cover map

**Fig. 2:**
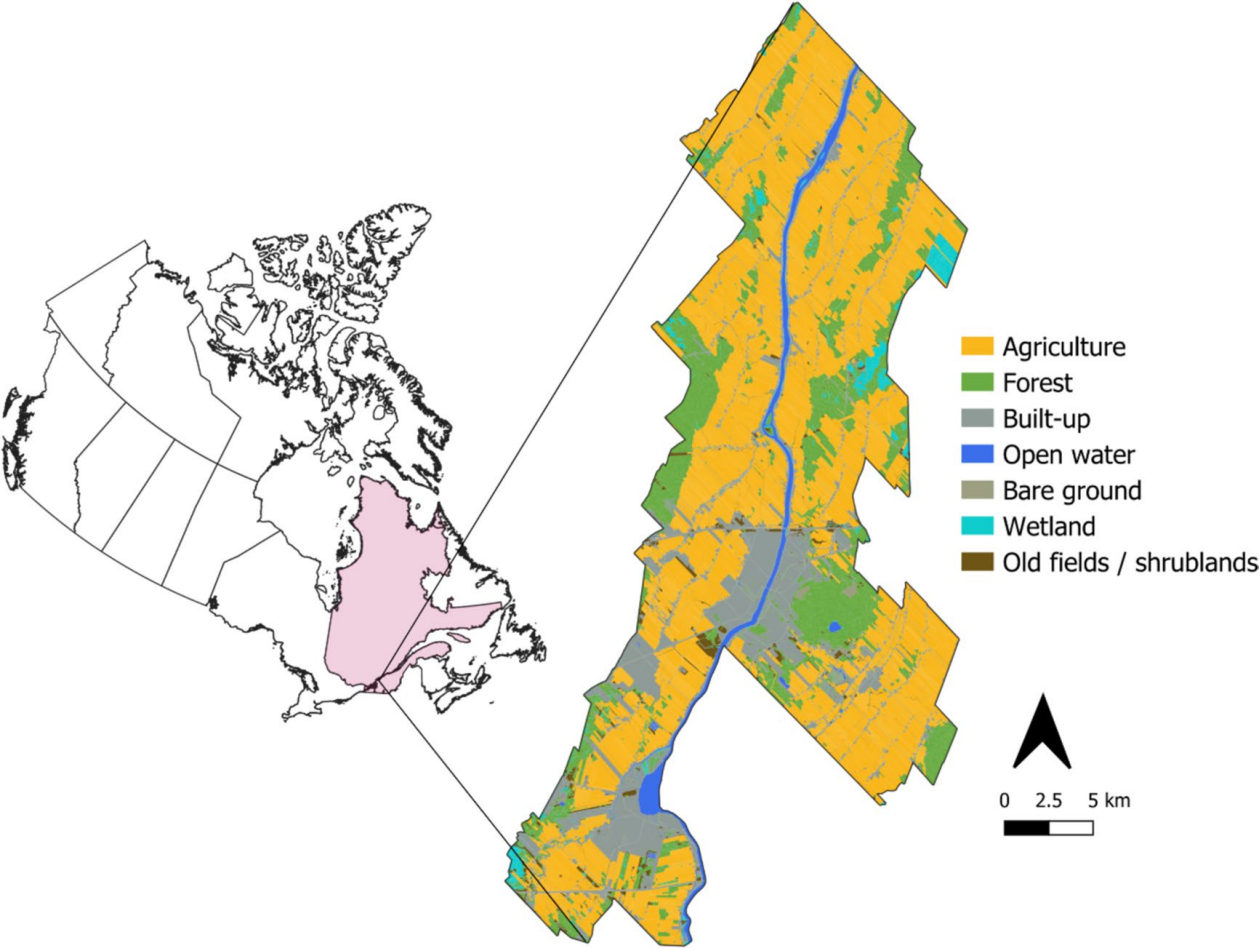
The county of La-Vallée-du-Richelieu is located in Southern Quebec (Canada) and extends over 60,449 ha. Eight major land use and land cover types are described: agricultural, forest, urban environment and roads (named built-up in the figure), open water, bare ground, wetlands, and old fields and shrublands. Land cover map sourced from ECCC et MDDELCC (2018)

The land use and land cover (LULC) map of La Vallée-du-Richelieu from the C*artography of Land Occupation in the Lowlands of the Saint-Laurent* (ECCC et MDDELCC 2018) identifies eight major LULC classes that depict the landscape as it was between 2013 and 2016 (Fig. 2). According to this map, the agricultural environment includes land categorized as cultivated and non-cultivated (e.g. ditches) and covers ~ 36,000 ha (~ 60% of LVR’s territory). Large forests, primarily mixed-deciduous, along with smaller forested patches and tree plantations, cover ~ 10,260 ha (~ 17% of LVR’s territory), and wetlands cover ~ 1,200 ha (~ 2% of LVR’s territory). The LULC category identified as *old fields/shrublands* (~ 1,880 ha, ~ 3% of LVR’s territory) includes areas covered by relatively low woody vegetation (~ 2 m height), shrubby vegetation, regenerating forest, etc. Some abandoned agricultural lands covered by pioneer vegetation are also included in this category. In Fig. 2, the categories named *human-modified environment* and *roads*, which cover ~ 9170 ha (~ 15% of LVR’s territory), were merged and identified as *built-up*. Bare ground and open water classes cover ~ 0.3% and ~ 3% of LVR respectively.

We further sub-classified some of the LULC types to obtain a more detailed map for the pollination supply model (see *Pollination provision* section below and Supplementary Information S1). The built-up environment was first reclassified into urban vegetation (e.g., urban parks or golf courses) or impervious surfaces (e.g., buildings or roads), and then reclassified by height (equal to and taller or shorter than 3 m) using LiDAR data, following the criteria by Communauté Métropolitaine de Montréal (2017). Wetlands were separated according to type (bogs, fens, peatlands, etc.). We also reclassified the old fields and shrublands class according to height (equal to and taller or shorter than 3 m). The agricultural class was reclassified into three subclasses: crops and cultures that potentially provide feeding resources for bees (e.g., berries, broccoli, tomatoes, etc.), crops that do not provide feeding resources for bees (e.g., wheat, sorghum, hay, etc.), and small non-cultivated elements (e.g., small paths, drainage channels, etc.). Cultivated and non-cultivated areas can be differentiated according to the information provided in the source LULC map (ECCC et MDDELCC 2018). For information on crop types cultivated on each field, we relied on the *Parcels and Reported Agricultural Productions* (PRAP) database (Financière Agricole du Québec 2023). The PRAP database consists of yearly polygon layers displaying the borders of crop fields that are declared for insurance purposes. Since crop field delimitation and number may differ slightly from year to year as farmland configuration changes, we used the PRAP 2014 polygon layer as our reference for the study region’s agricultural area configuration and composition. In this way, we aligned our maps and results with data in the official LULC map (ECCC et MDDELCC 2018). Importantly, the crop fields in the PRAP database (2014) cover ~ 33,700 ha (i.e., 2,300 ha less than the cultivated region as informed in the LULC map). This difference is likely attributable to the different data sources used to build the PRAP database and the LULC map. For all our purposes, we prioritized the PRAP database and thus considered that the agricultural zone covered 33,700 ha. For analysis purposes, we converted the LULC vectorial map into a 30-m resolution raster. More details are available in Supplementary Information S1.

All maps in this research were generated using QGIS (QGIS 2024). Spatial analysis and other treatment of spatial data were performed using QGIS (QGIS 2024) or R (R Core Team 2018). The projection was NAD 83/Quebec Lambert EPSG: 32198 for all maps. Raster maps had a final resolution of 30 m. For spatial analysis, maps included a 2-km buffer around the study region’s border (not shown in Fig. 1) to avoid edge effects which could lead to underestimation of pollinators’ abundance.

### Restoration targets

With the overarching goal of enhancing wild-bee pollination provision without compromising crop production in LVR, we set the following *lower-threshold targets* for restoration: (i) All crop fields must have a minimum of 20% natural habitat coverage within 1 km from fields’ center (assuming that 20% is the minimum amount of natural habitat within the vicinity of crops that ensures wild-bee populations persistence and pollination provision); and (ii) wild-bee pollination provision is ensured for the agricultural area of the study region.

#### Identification of marginal agricultural fields

Following our framework, we next identified all the marginal agricultural lands, i.e. land with presumably less value for crop production that could be restored to natural habitat. In our case, marginal lands included all abandoned and degraded agricultural fields, as well as the longest border of each crop field.

#### Identification of abandoned fields

To identify abandoned agricultural fields, we used the cessation of farming for a minimum of 5 years as our criterion to assess abandonment (Anguiano et al. 2008). A five-year time lapse is also suitable to avoid potential false positives from fallow practice. To identify unused crop fields, we used maps for the period 2012–2021 from the PRAP database described in the section *La Vallée-du-Richelieu's land use and land cover map*. We converted these maps into 30-m binary rasters, in which pixel values indicated the cultivation status (cultivated or not) of the corresponding field each year. We then stacked the yearly raster maps, and searched for those fields which were not cultivated for at least five years, i.e., from 2017 to 2021. We did not include former crop fields in the *old fields and shrublands* category on our abandoned fields map, since we assumed that these fields are already covered with woody vegetation. More details and limitations of our approach are available in Supplementary Information S2.

#### Identification of degraded fields

To identify the potential degradation of crop fields, we used maps of the Normalized Difference Vegetation Index (NDVI) of the study area and looked for sectors where the NDVI has declined over time as a proxy for vegetation decline (Bai et al. 2008; Dubovyk et al. 2013). NDVI has been used to measure crop yields (Rieb and Bennett 2020; Shen and Evans 2021) considering that the profile of the NDVI over a growing season follows the crop cycle: NDVI increases as crops grow, then peaks during the crops’ most productive stage, and decreases after harvest (Huang et al. 2014). An overall decreasing trend in the NDVI over multiple cycles of crop production could be indicative of declining crop yields. Even though crop yields can decline for multiple reasons (e.g. drought, pests, etc.), including agricultural intensification itself (Burian et al. 2024), such a decline can be a potential indicator of soil degradation (Dubovyk et al. 2013; Easdale et al. 2019). To measure NDVI, we acquired Landsat 5, 7, and 8 satellite images (surface-reflectance corrected dataset) that covered the study region, using the Google Earth Engine platform (Gorelick et al. 2017). We performed a series of image manipulation to improve image quality. First, the collection of satellite images was filtered to include only images from June to September for the period from 2000 to 2021. We expected that a 22-year period would be long enough to assess a general trend in NDVI. We then removed clouds from images. To ensure NDVI trends were not influenced by soil effects or land cover types (e.g., forests, roads) on the edges of crop fields we removed edge pixels (25 m) along the border of crop fields (Montandon and Small 2008). Next, we computed the median NDVI value across a growing season to obtain the annual NDVI and performed a pixel-wise linear regression of the NDVI versus year, using the *lm* function from the *stats* package (R Core Team 2018). We sub-selected those pixels showing a significant (p ≤ 0.05) negative NDVI trend. Most patches that showed a negative trend in the NDVI covered only a portion of the crop fields area and were located at disparate locations within farms. We also obtained a number of individual pixels (30-m resolution) showing a negative NDVI trend over time. Although restoration of small patches of habitat inside crop fields might be beneficial from an ecological perspective (Bodin et al. 2006; Brosi et al. 2008; Riva and Fahrig 2022), it could be less acceptable and feasible from the landowner’s perspective (for instance, a restored patch misplaced within a crop field can interfere with machinery operation). For that reason, and for simplicity, we removed patches with negative NDVI trend smaller than 0.15 ha and we only retained those fields in which patches of degraded land covered ≥ 30% of the crop field area and assumed that the entire field was potentially degraded; otherwise, the field was considered to be non-degraded. More details and limitations of our approach are available in Supplementary Information S3.

### Pollination provision

#### Pollination supply

We modeled pollination supply using the software InVEST® 3.3.0 (Natural Capital Project 2024). The InVEST model for crop pollination estimates the relative abundance of wild bees visiting each cell in the landscape, based on the availability of nesting habitat and feeding resources surrounding the cell, as well as the flying range of pollinators (Lonsdorf et al. 2009). As for the bees modeled, we used ten wild-bee genera identified in the study region by Botzas-Coluni et al. 2021 (Table S1). The model requires that the nesting preferences (cavities, soil, etc.), the seasonal feeding activity and the flying ranges are set for each genera modeled. We based our parameterization in literature data for the genera used (Packer et al. 2007; Zurbuchen et al. 2010; Agriculture and Agri-Food Canada 2014; Zhao et al. 2019; Harmon-Threatt 2020; Natural Capital Project 2024). Because of lack of empirical data and because we were mainly interested in enhancement of habitat for wild bees, we did not consider the use of managed bee colonies in our model, which can lead to underestimating pollination supply. The model also requires data on the suitability of land cover types to provide nesting habitat and feeding resources to wild bees. We parameterized the model based on literature-derived estimates (Schulp et al. 2014; Koh et al. 2016; Groff et al. 2016; Zhao et al. 2019; Fernandes et al. 2020). Because of a lack of empirical data in our study region to set accurate parameters to our model, we chose to assign the values 0, 0.25, 0.5, 0.75 and 1, indicating relative suitability. For instance, a value of *zero* means no nesting capacity or no floral resources availability for a land cover type, while a value of *one* means highest nesting capacity or floral availability, relative to the other land cover types (Tables S1, S2). We had our model parameters verified by six independent bee experts, whose opinion was used to adjust final values. The model produces two maps of bee abundance, one for the spring season and another for the summer season. Since the seasonal aspect of pollination was beyond the scope of our research, we used the summer abundance map only.

The InVest model of crop pollination produces a map of relative bee abundance. This imposes some limitations, namely the impossibility of mapping absolute pollination supply. However, the relative bee abundance is still suitable for making qualitative comparisons among land-use change scenarios, which is ultimately our objective (see Crop Pollination model user’s guide in Natural Capital Project 2024). More details are available in Supplementary Information S5.1.

#### Pollination demand

Pollination demand was mapped for each field as the area covered by pollination-dependent crops weighted by the relative level of pollination dependency of each crop type (Schulp et al. 2014; Koh et al. 2016). Pollination dependency levels were derived from Klein et al. (2007) and Mcgregor (1976), and information on each crop type and field size was obtained from the PRAP database. Since more than one crop type is cultivated in some fields, we used the main crop, based on the PRAP database, to define the field’s pollination dependency. In addition, since crops are rotated in the study area, we mapped pollination demand on each field between 2012 and 2021, and then obtained the average pollination demand on each crop field over this 10-year period. We assumed that crop fields previously identified as abandoned or degraded had no need for pollinators, at least for food production. More details are available in Supplementary Information S5.2.

#### Spatial match and mismatch between pollination supply and demand

We qualitatively assessed the spatial match-mismatch between pollination supply and demand in the study region using choropleth bivariate maps (Schulp et al. 2014; Koh et al. 2016; Hemberger and Gratton 2023). We first classified pollination supply and demand into three levels (low, intermediate, and high) using tertile classification (*quantile* function from the *terra* package, (R Core Team 2018), and then built a bivariate map to assess the spatial overlap between supply and demand levels. Bivariate maps allow for rapid visualization of areas where pollination supply and demand levels are disparate. For example, in areas where supply is relatively low and the demand is relatively high, pollination provision is probably not attained, and management interventions would be a priority. We defined crop fields where demand was intermediate to high and supply was low as *hotspots for pollination demand*.

### Natural area coverage

The natural area coverage in agricultural landscapes positively correlates with the abundance of within-field pollinators (Raderschall et al. 2021) and, likely, with pollination supply and crop productivity (González-Chaves et al. 2022). To compute the natural area coverage around crop fields, we first calculated the per-pixel natural area percentage within a 1-km buffer, and then we calculated the average natural area percentage within 1-km from each field’s centroid. A distance of 1 km is commonly used to evaluate pollination-related metrics (Garibaldi et al. 2011; Klein et al. 2012; Botzas-Coluni et al. 2021). Calculations were performed using the *focal* function from the *terra* package in R (R Core Team 2018). We considered natural areas to include all land classified as forests, wetlands, old fields and shrublands, and flower strips. Due to a lack of detailed information on existing semi-natural habitats like hedgerows or windbreaks, the actual natural area percentage may have been underestimated for the study region.

### Restoration scenarios

We created four restoration scenarios considering different practices that are applicable in agricultural landscapes, and one baseline scenario for comparison purposes (Table 1). These five scenarios were conceived to evaluate the possibility of meeting the lower-threshold targets that we defined (section *Restoration targets*), within the limits imposed by land availability in LVR. The *no restoration* scenario served as a baseline to evaluate the spatial match-mismatch between pollination supply and demand, and the natural area coverage in the study region. The *reforestation in abandoned and degraded fields* scenario served to evaluate the benefits of creating forest patches in all abandoned and degraded fields in LVR (this corresponds to 3% of the territory, see Supplementary Information S4). Forest patches are mostly suitable as nesting habitats for wild bees, as feeding resources are scarcer in this type of habitat. The *flower strips on edges* scenario was conceived to evaluate the establishment of flower strips (covering 3% of the study area) on the longest edge of crop fields. Flower strips provide feeding resources and attract wild bees to the vicinity of fields. The fourth scenario, named *mixed strategy*, served to evaluate the combined effect of establishing flower strips on the longest edge of some fields and reforestation in some of the all the abandoned or degraded fields. To ensure that the *mixed strategy* scenario would have the same amount of area restored as the other scenarios (3% of the study region’s territory), we performed a simple prioritization exercise to select from all potential flower strips that could be established and all the abandoned and degraded fields that could be reforested. First, we identified crop fields in the *no restoration* scenario that had low pollination supply and intermediate to high demand (i.e., the hotspots for pollination demand). From among these, we selected fields that had, on average, less than 5% natural area coverage within 1 km from the center, as this can be considered a simplified landscape. We expect that these fields will have greater gains from restoration than fields with higher amounts of surrounding natural area (Tscharntke et al. 2005; Klein et al. 2012). Once the crop fields to prioritize for restoration of natural habitat in their surroundings were selected, we defined the buffer area where restoration should be performed so these fields could benefit from pollination services. We first drew a circular buffer with a 1-km radius from the centroid of each priority field. We then selected all flower strips and abandoned and degraded lands that intersected with this buffer area. We gradually removed flower strips located at increasing distances from abandoned and degraded fields (assuming that no flower strips would be planted close to a forested patch), until we achieved the target of restoring 3% of the study area. After this prioritization, the *mixed strategy* scenario represented ~ 891 ha of abandoned and degraded lands reforested and ~ 968 ha of flower strips planted. A fifth scenario, named *maximal restoration*, in which all the abandoned and degraded fields and the longest edge of most crop fields would be restored, was conceived to evaluate the limits that land availability imposes on restoration outcomes. This scenario represented ~ 5.8% of LVR’s territory. All scenarios were built by reclassifying the current LULC map of LVR using the *reclassify* function from the *terra* package in R (R Core Team 2018). More details are available in Supplementary Information S4.

**Table 1:** Summary of the restoration scenarios evaluated, including the area restored. The scenarios *reforestation in abandoned and degraded fields*, *flower strips on edges* and *mixed strategy* simulated that 3% of LVR’s territory would be restored, while the scenario *maximal restoration* simulated 5.8% of LVR restored

Scenario	Description	Area restored (% of LVR’s territory)
No restoration	Current LULC. No restoration interventions considered	0 ha
Reforestation in abandoned and degraded fields	Trees planted in all fields identified as abandoned or degraded	~1,830 ha (3%)
Flower strips on edges	A 5-m wide flower strip planted along the longest edge of each crop field (except in those identified as abandoned and degraded)	~1,826 ha (3%)
Mixed strategy	Prioritized abandoned and degraded lands reforested, in combination with the establishment of a 5-m wide flower strip on the longest edge of selected crop fields	~1,858 ha (3%)
Maximal restoration	All the abandoned and degraded lands reforested, a 5-m wide flower strip established on the longest edge of each crop field (except for those within 50 m of fields identified as abandoned and degraded)	~3,478 ha (5.8% total: 3% reforested and 2.8% as flower strips)

### Scenario comparison

We assessed the extent to which the lower-threshold targets were achieved in the four restoration scenarios (Table 1) by comparing the enhancement of pollination provision and natural area coverage under each scenario to the baseline scenario.

#### Enhancement of pollination provision

To compare the relative effectiveness of the alternative restoration scenarios in enhancing pollination provision, we analyzed the increase in supply relative to demand using the supply-to-demand ratio (S/D). First, we mapped pollination supply for every scenario, using InVest software as described in the *Pollination supply* section above. Pollination supply raster maps (for every scenario) and the demand raster map (which was assumed to be the same for all scenarios) were subsequently standardized between 0 and 1 by linear scaling (min max normalization) (Zhao et al. 2019). We then calculated the ratio between the scaled supply and demand (S/D) for each scenario, for fields with demand > 0. This approach relies on some assumptions, and consequently has important limitations worth mentioning here. The scaling of supply and demand, and their ratio, might lead to a misinterpretation of these variables, mainly an assumption that one unit of pollination supply is equivalent (i.e. fulfills) one unit of pollination demand. This interpretation would not be correct given the nature of the proxies used to map supply and demand in this article, which refer to different aspects of the pollination process. More precisely, it is not possible to claim that a given value for relative pollinators abundance (supply, S) will effectively pollinate a given area of pollination-dependent crop (demand, D). Then, a straightforward and uncautious interpretation of the S/D assumes that a S/D ≥ 1 is indicative of supply levels being equal or higher than demand levels, i.e., a supply that complies (S/D = 1) or exceeds (S/D > 1) the demand. For the same reason stated before, it would be incorrect to derive this interpretation of the S/D metric in our case, since the S/D metric here does not provide an absolute measure of pollination deficit, balance or surplus, (Schulp et al. 2014; Koh et al. 2016; Zhao et al. 2019; Fernandes et al. 2020). In our case, the S/D metric is a way to qualitatively compare the relative effectiveness of scenarios in enhancing pollination supply relative to demand. Under this interpretation, since demand remains immutable across scenarios, a larger value for the S/D in a given scenario implies a larger increase in the supply relative to the demand for that scenario, as compared to another scenario (including the baseline) with a lower S/D. Certainly, we could have used only the difference in the supply after restoration to compare scenarios, but we were interested in the enhancement of supply within crop fields relative to their demand.

We aggregated the S/D by calculating its mean value at 1 km^2^. To do that, we created a grid of 1-km^2^ cells with the extent of the LULC map and retained only those cells that overlapped at least 25% with the agricultural region. We then calculated the percentage of the 1-km^2^ cells with a S/D ≥ 1. We repeated this procedure for each one of our five scenarios.

#### Restoration priorities based on natural area coverage

Given the importance of natural habitat to sustain pollination provision, crop fields that have little natural habitat in their vicinity should be prioritized for restoration. To compare the five restoration scenarios described above in terms of natural area coverage around crop fields we built a restoration priority ranking. For each scenario, we classified all crop fields into one of the four following categories according to the level of restoration priority: (a) High priority: fields with less than 5% surrounding natural area, (b) Intermediate priority: fields with 5% or more but less than 10% surrounding natural area, (c) Low priority: fields with 10% or more but less than 20% surrounding natural area, and (d) No priority: fields with 20% or more natural area in their surroundings. We then summed the total area of crop fields in each category, for each scenario, and calculated the percentage of the agricultural zone that fell into each category.

## Results

### Abandoned and degraded agricultural fields

**Fig. 3:**
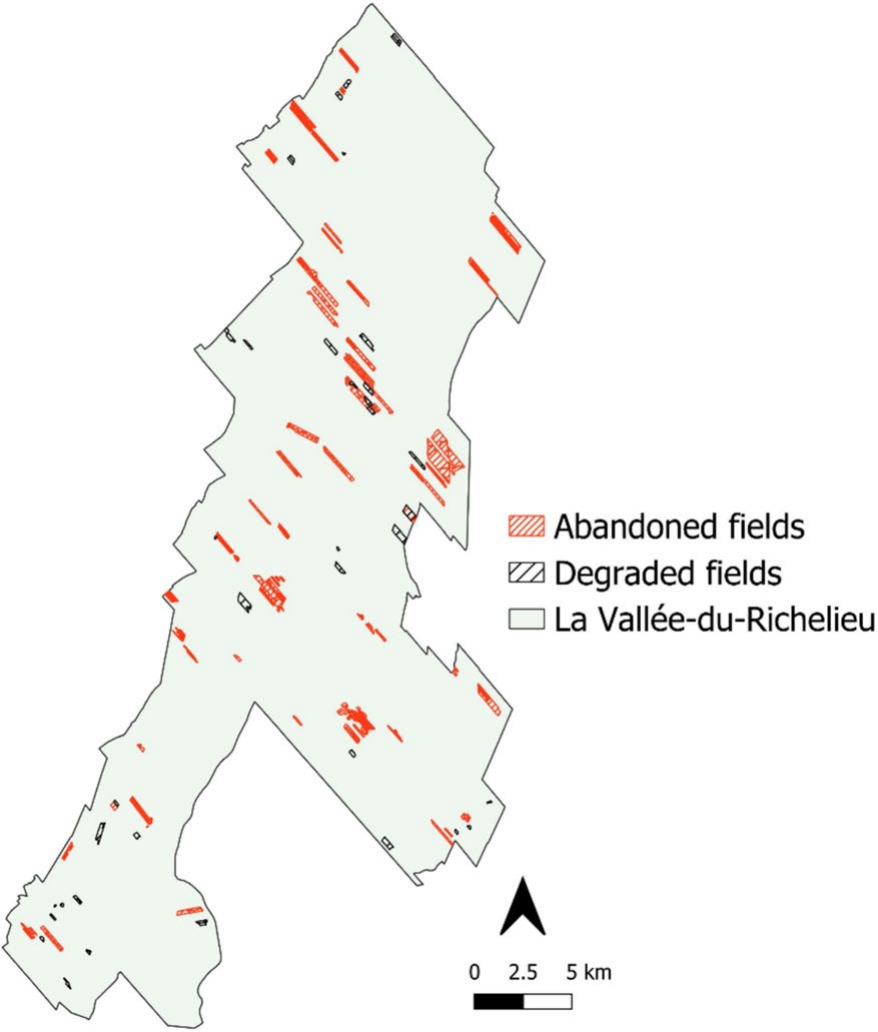
Crop fields not declared for agricultural use for five consecutive years up to 2021 (the final year within the scope of this study) were classified as abandoned (orange polygons), and cover over 1,550 ha. Fields with more than 30% of their area showing a negative trend in the NDVI for a 22-year period (2000–2021), were classified as degraded (black polygons), and extend over 312 ha

We identified 1,550 ha of abandoned crop fields, and 312 ha of crop fields with large proportions (≥ 30%) of degraded soil (Fig. 3). Together, abandoned and degraded fields comprise 3% of La Vallée-du-Richelieu’s (LVR) territory, or 5.5% of its agricultural zone.

### Enhancement of pollination provision

#### Baseline for pollination supply and demand

**Fig. 4:**
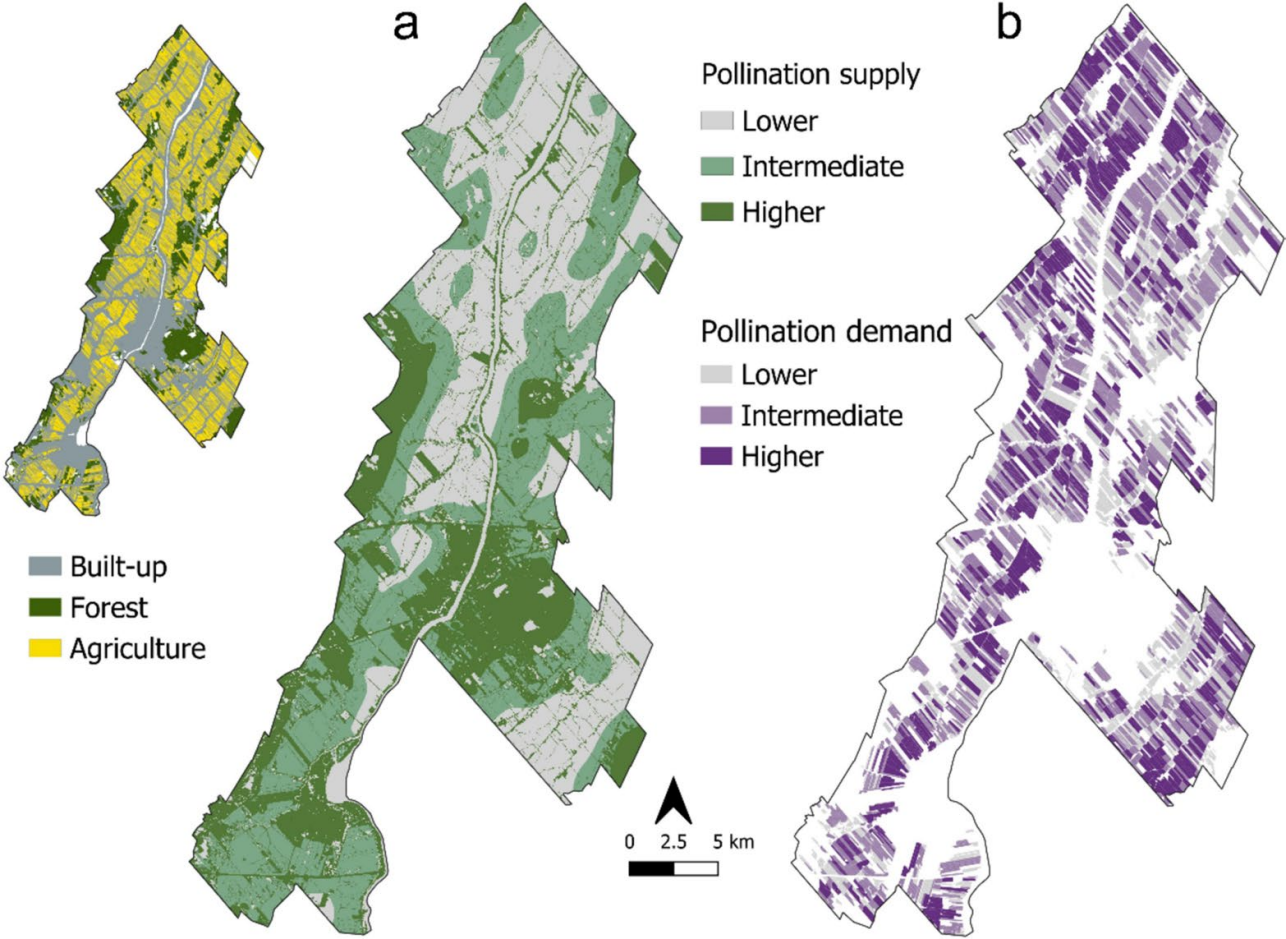
Pollination supply was classified into areas of low (gray), intermediate (light green) and high (dark green) supply (**a**). The inset image in the upper left shows the three larger LULC types in the study area: built-up, forest, and the agricultural zone. Zones with intermediate and higher pollination supply overlapped mostly with built-up environment and forested areas, and to a lesser extent with the agricultural zone, mainly in the South of the study region. Pollination demand was also classified into zones of low (gray), intermediate (light purple) and high (dark purple) classes, and was intermediate to high throughout the cultivated region (**b**)

Although the normalized pollination supply index (ranging 0–1) was low overall (mean: 0.1 ± 0.1), we divided our pollination supply map into areas with low, intermediate, and high supply. Pollination supply values were higher in built-up (urban) areas (dark green in Fig. 4a), with 47% of the high-pollination-supply zone overlapping with the built-up area. Other zones with high pollination supply overlapped with forests (33% of the high-supply zone), while 11% of the high-supply zone overlapped with the agricultural environment. Most of the agricultural zone (95%) presented low to intermediate levels of pollination supply. The normalized crop demand index had an average of 0.15 ± 0.16 (mean ± SD). Pollination demand was intermediate to high over the entire agricultural region of the study area (Fig. 4b).

**Fig. 5:**
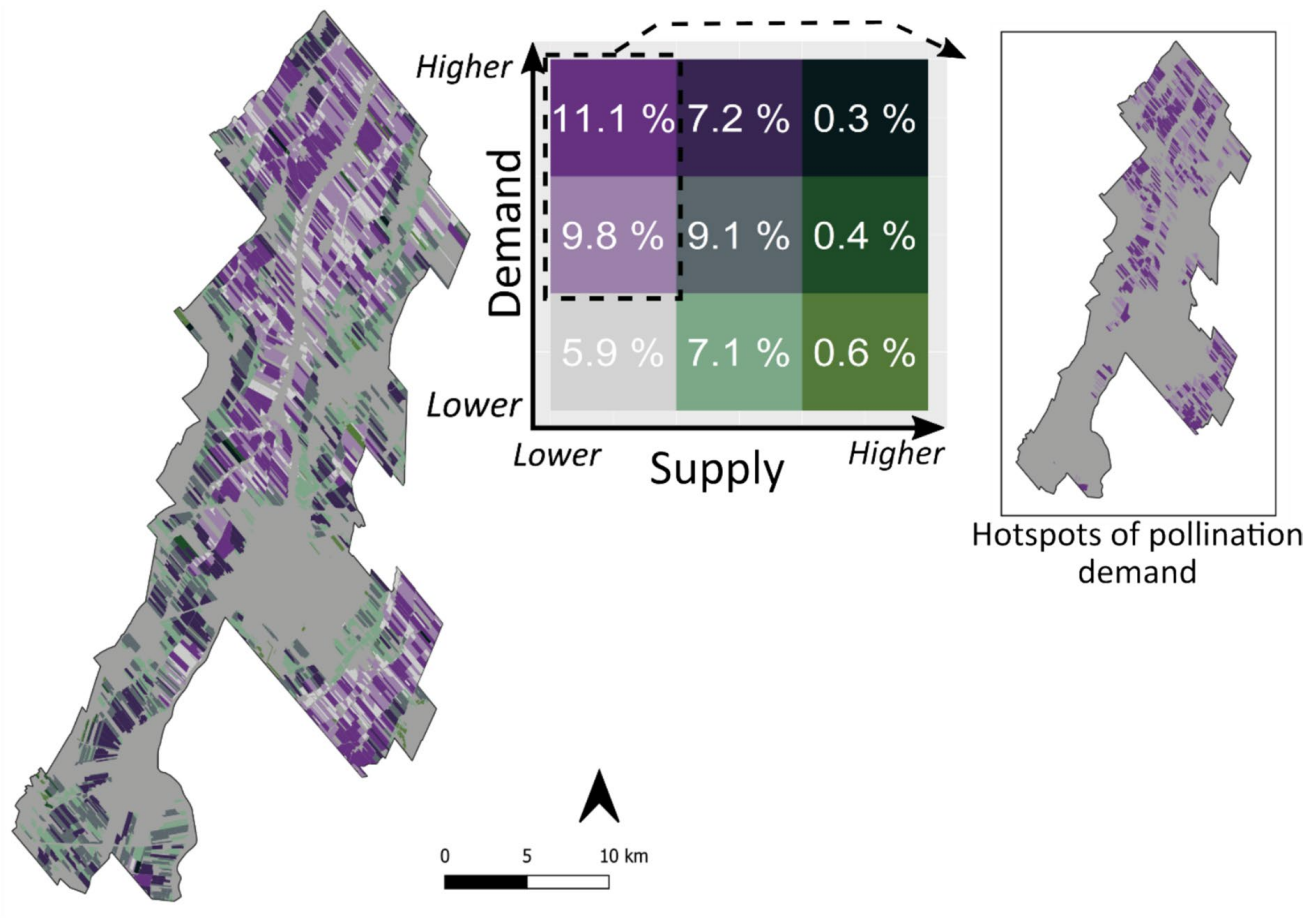
Pollination supply and demand maps, classified in tertiles, were overlaid to display the spatial correlation between these variables at the field scale. This analysis produced a matrix (middle top), with nine levels of correspondence between pollination supply and demand, from low supply and low demand in the lower left corner, to high supply and high demand in the upper right corner. The percentage of the study region that fell into each category is shown in the matrix. The top left cells in the matrix indicate the hotspots for pollination demand, i.e., fields with intermediate to high demand and low (or no) supply. The location of the hotspots for demand are shown in the inset image to the right

The overlap of supply and demand maps (Fig. 5) showed that zones with higher levels of pollination provision cover only 1.3% of the total cultivated area (Fig. 5, bivariate matrix), regardless of the demand level. Hotspots for pollination demand, i.e., crop fields where relative levels of demand are intermediate to high and relative levels of supply are low, make up for almost 21% of the cultivated area (Fig. 5 inset).

#### Pollination provision enhancement through restoration

**Fig. 6:**
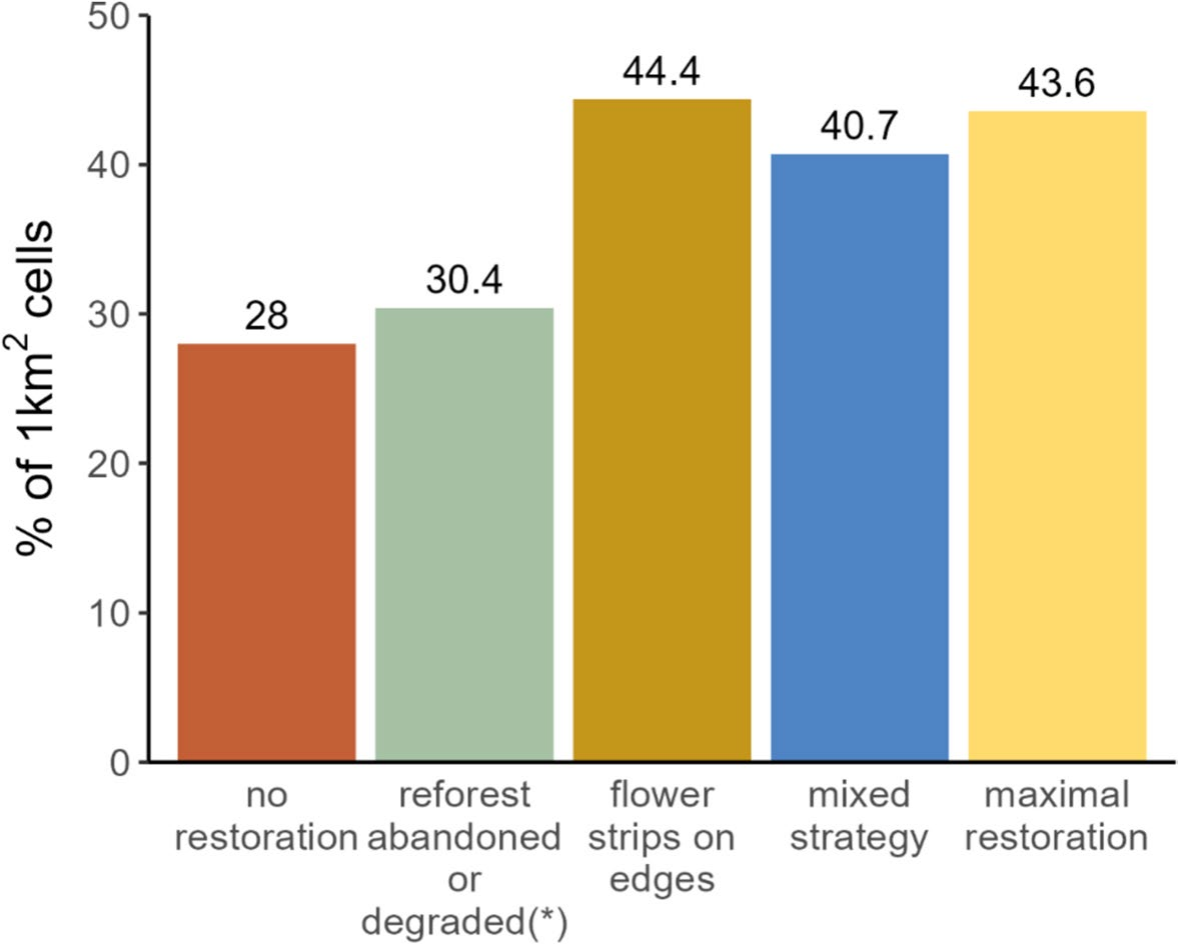
The efficiency of the different restoration scenarios in enhancing pollination provision was compared by aggregating the supply-to-demand ratio (S/D) to its mean value at 1-km^2^. In the bar plot, the Y-axis represents the percentage of 1-km^2^ that had a mean S/D ≥ 1. The best performing scenarios are those that included flower strips, yet more than half of the cultivated area had a S/D < 1, suggesting persistent deficits in wild-bee pollination. (*) “*Reforestation in abandoned and degraded fields*” scenario

The relative enhancement of pollination provision among scenarios was assessed by computing the supply-to-demand ratio (S/D) in the agricultural region, aggregated at 1-km^2^ cells. In the *no restoration* scenario, 28% of the 1-km^2^ cells had a S/D ≥ 1. The different restoration scenarios made it possible to increase this ratio by 2.4% in the *reforestation in abandoned and degraded fields* scenario, by 16.4% in the *flower strips on edges* scenario, by 12.7% in the *mixed strategy* scenario, and by 15.6% in the *maximal restoration* scenario (Fig. 6).

### Natural habitat coverage and restoration priorities

**Fig. 7:**
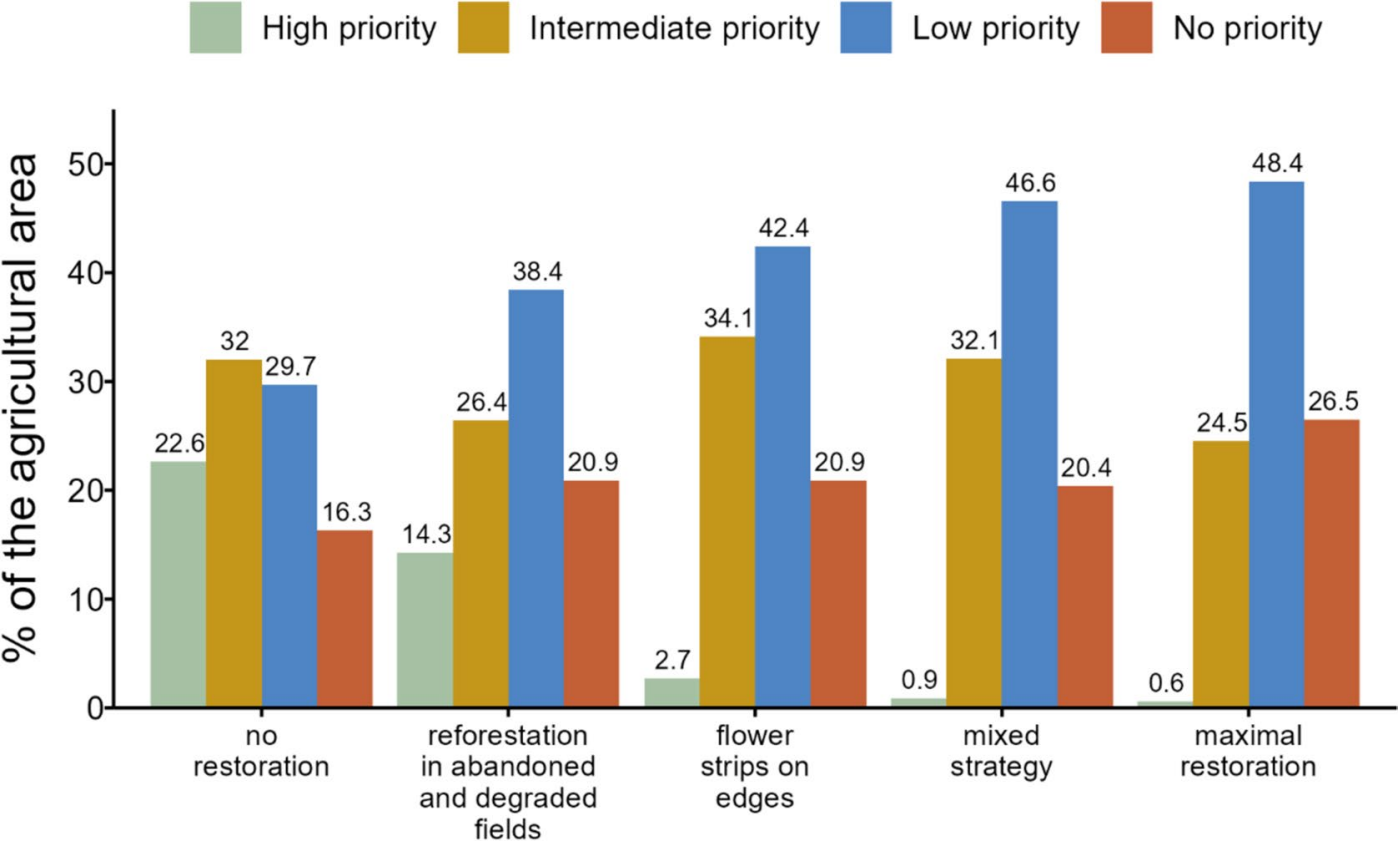
Restoration priority ranking based on the amount of natural habitat surrounding crop fields. The restoration priority ranking consists of five categories: *High priority*, when there is 5% or less natural habitat coverage within 1 km from the center of the field; *Intermediate priority*, between 5 and 10% natural habitat coverage; *Low priority*, more than 10% and less than 20% natural habitat coverage and *no priority*, 20% or more natural habitat coverage. First, we calculated the total area of crop fields in each category, and then the percentage of agricultural area (a total of 33,700 ha) in each rank category, for each scenario

We also compared how the different restoration scenarios increased the area of natural habitat surrounding crop fields, and assigned restoration priorities according to natural habitat deficits. In the baseline–the *no restoration* scenario–, 22.6% of the agricultural zone of LVR ranked as a high priority area for restoration (red bars in Fig. 7), i.e., ~ 7,618 ha of crop fields had < 5% natural habitat within 1 km. Restoration of marginal lands alone helped to reduce this shortfall in natural habitat to 14.3%, 2.7%, 0.9%, and 0.6% in the *reforestation in abandoned and degraded fields*, the *flower strips on edges*, the *mixed strategy*, and in the *maximal restoration* scenarios, respectively. Most of the agricultural region changed from high priority for restoration (red bars) to low priority (blue bars). The agricultural area with > 20% natural habitat within 1 km from crop fields (*no priority* category, light blue bars in Fig. 7) increased from 16.3% in the *no restoration* scenario to nearly 20% in the *reforestation in abandoned and degraded fields*, the *flower strips on edges* and the *mixed strategy* scenarios, and up to 26.5% in the *maximal restoration* scenario. Still, almost 73% of the agricultural area did not achieve the 20% lower-threshold target for natural habitat coverage in the *maximal restoration* scenario.

## Discussion

We propose a simple methodological framework for assessing the feasibility of meeting lower-threshold restoration targets within area constraints in agricultural landscapes. We showed that lower-threshold targets could not be met if only marginal agricultural areas are restored in our study area. However, most of the crop fields that currently have minimal amounts of surrounding natural habitat (< 5%, which is typical in homogeneous landscapes (Tscharntke et al. 2005)) were able to increase it to higher amounts (between 10 and 20%) in all restoration scenarios. Our framework was also useful for identifying priority fields for restoration of natural habitat in their surroundings. Our findings suggest that there is significant potential to increase the multifunctionality of the agricultural landscape of the study region with no detriment of crop production. Besides marginal lands restoration, there are multiple practices that can complement and even synergize with remnants of natural areas to boost and sustain biodiversity within cropland and that have minor immediate impact on crop production, such as cultivating in smaller fields (for the same total area cultivated), increasing crop heterogeneity, creating small patches of native vegetation, and less intervened edges (Blaauw and Isaacs 2014; Fahrig et al. 2015; Sirami et al. 2019).

A restoration strategy based only on the reforestation of all the abandoned and degraded fields in the study area showed only a 2.4% increase in pollination provision (evaluated by the relative change in the S/D) at the landscape scale. The limited increase in pollination provision in the *reforestation of abandoned and degraded lands* scenario (as compared to the *no restoration* scenario) may be attributed in part to the spatially clustered pattern of abandoned and degraded fields (data not shown), which is also typical of agricultural settings (Su et al. 2018; Wei et al. 2021; Li et al. 2021). It can be also attributed to the fact that wooded areas mainly increase the availability of nesting sites for wild bees, while foraging resources are less abundant in forests. While we did not focus our research on the specific needs of wild bees in terms of nesting or feeding habitat, the InVest pollination model does consider the nesting and feeding capacity of each land cover type. In our model parametrization, forested areas provided better nesting capacity than flower strips (see Table S2 in Supplementary Information S5.1). The reforestation strategy performed better when coupled with the establishment of flower strips on edges, which has been shown to increase in-field pollinator abundance by providing foraging resources for wild bees in the vicinity of crops (Morandin and Kremen 2013; Albrecht et al. 2020; Shi et al. 2021). Scenarios with the same total area restored but that included flower strips (*flower strips on edges* and *mixed strategy*) produced greater increases in pollination provision. This outcome can be attributed to the fact that flower strips were situated close to crop fields and were more evenly distributed across the entire region than reforested patches. Our parametrization of the pollination supply model also explains in part this result, since we considered flower strips as a more favorable feeding resource for wild bees than forests (see Table S2 in Supplementary Information S5.1), while they also provide some nesting resources for ground nesters. The *maximal restoration* scenario produced similar results to the *flower strips on edges* and the *mixed strategy* scenario regarding pollination provision at the landscape scale. Our results suggest that patches of nesting habitat combined with smaller patches of feeding resources interspersed throughout the agricultural matrix enhance pollination provision, as suggested by previous studies (Brosi et al. 2008; Kremen and M’Gonigle 2015; López-Cubillos et al. 2023).

We believe it is pertinent to add a brief digression on our use of the S/D as a metric to evaluate pollination provision. As we claimed in the *Methods* section, the S/D cannot be used to assess the actual extent of pollination demand fulfillment, for the reasons explained in that section. It follows that we cannot ensure that a S/D = 1 means that the pollination supply achieved through restoration matches the demand, nor can we say that a S/D > 1 means a surplus of pollination. In that sense, we must be really careful on the interpretation of our results in Fig. 6. As shown by the empirical research, larger amounts of natural habitat will lead to an enhanced abundance and diversity of pollinators within crop fields, and consequently we can expect higher pollination activity and higher crop yields. That evidence is ultimately the rationale for relying on the S/D metric as a measure of the relative effectiveness of scenarios in enhancing pollination supply relative to demand. That is, we expect that a higher S/D after restoration will correlate with higher pollinator abundance and pollination provision. Even with this limitation, we believe that our analysis is useful to explore and compare among potential restoration strategies regarding the possibility of enhancing pollination.

Our approach could be improved by fine-tuning both the composition of restored sites, to take into account wild bees’ dietary needs (Tissier et al. 2023), and the configuration of restored areas, to ensure the resulting network includes sufficient nesting sites and feeding resources for wild bees (Brosi et al. 2008; López-Cubillos et al. 2023). Tailored restoration schemes could be developed to also favor other species and ecosystem services (Kremen and M’Gonigle 2015). In addition, other marginal areas could be considered for restoration such as riparian zones, slopes, eroded areas, and even elements of infrastructure like roadsides, railway banks, powerline right-of-way, and waterways (Gilbertson et al., n.d.). A spectrum of restoration strategies and targets, over a gradient of land-sharing/land-sparing strategies (Law et al. 2015, 2017), could also improve our approach, considering that a transition to landscape multifunctionality is likely to occur gradually. The suitability of scenarios and targets should also be evaluated and defined in close collaboration with stakeholders (Metzger et al. 2017).

The methodological framework presented proved useful for demarcating the limits for restoration benefits within an agricultural landscape. These restoration limits refer to a *ceiling*–an upper limit– for achieving targets, given the constraints that are imposed by trade-offs between crop production and provision of non-crop ES (Law et al. 2015). We intentionally set aside economic, legal, and practical aspects of restoration for the sake of the theoretical exercise. While our results are specific to the study region considered, similar outcomes can be anticipated in other intensive agricultural landscapes, where land is mostly used for crop production and so land available for restoration is scarce. In fact, given the common pattern of relationships between biodiversity, ES, and production trade-offs in commodity-production landscapes, we believe our framework can be readily applied in contexts other than agriculture and to evaluate the potential to enhance other ES.

Our results appear relevant in the particular context of the region where our study area is located (the Montérégie region of Québec), where the majority of the land abandoned is left to spontaneous forest regeneration (Institut de la Statistique du Québec 2024). Loss of agricultural land is a concern in this region, but if properly managed, it could also be a feasible opportunity to restore natural habitat and transition to a more sustainable agriculture.

Overall, our results suggest that it is feasible to balance crop production and restoration of natural habitat (and thus increase pollination by wild bees) in the initial stages of a transition towards more multifunctional agricultural landscapes. Nonetheless, to meet more ambitious targets such as the Kunning-Montreal Global Biodiversity Framework Target 2 (30% of degraded areas to be restored by 2030), substantial changes in modern crop production systems may be needed.

## Conclusion

Agricultural expansion is one of the main drivers of land change worldwide (Foley et al. 2005, 2011). Restoration is expected to bring multiple benefits in terms of biodiversity recovery and ecosystem services provision, but it is a challenge in productive landscapes such as croplands, given land availability constraints. Restoration of marginal lands is certainly a potential compromise solution, and in that direction, we highlight the practicality of our framework to evaluate the potentials and limits for restoration in agricultural landscapes. Our study showed that restoration of marginal lands alone can bring benefits during the initial stages of a transition towards more multifunctional agricultural landscapes but is a limited approach in the long term. The relevance of our framework is also grounded on the claims for more specific and tailored target definition when planning for restoration, especially when broad and global targets are to be downscaled to the regional and local scales (Jørgensen 2015; Gilby et al. 2021). We expect that our framework will help researchers and planners to characterize agricultural landscapes and determine which restoration targets are realistic in the transition towards landscape multifunctionality.

## Supplementary Information

Below is the link to the electronic supplementary material.

Supplementary file1 (DOCX 36 KB)

## Data Availability

The datasets generated and R scripts are available from the corresponding author upon request.
