# Supplementary material for: From marginal croplands to natural habitats: A methodological framework for assessing the restoration potential to enhance wild-bee pollination in agricultural landscapes
Source: Landsc Ecol. 2024 Nov 12;39(11):194. doi: 10.1007/s10980-024-01993-y (PMC11554958; doi:10.1007/s10980-024-01993-y)
Supplement: Supplementary file 1 — Supplementary file1 (DOCX 36 KB) [file 10980_2024_1993_MOESM1_ESM.docx]

**Supplementary Information**

**From Marginal Croplands to Natural Habitats: A Methodological Framework for Assessing the Restoration Potential to Enhance Wild-Bee Pollination in Agricultural Landscapes**

**S1. Reclassification of the land use and land cover map of La Vallée-du-Richelieu**

The land use and land cover (LULC) map of La Vallée-du-Richelieu (LVR) [(ECCC et MDDELCC, 2018)](https://www.zotero.org/google-docs/?sdvloH) served two main purposes. First, it was used as an input for the InVest^®^ [(Natural Capital Project, 2024)](https://www.zotero.org/google-docs/?kLkb3C) crop pollination model. Second, it was used to build the *no restoration* scenario, i.e., to describe the baseline or current situation in the study region.

In applying the InVest crop pollination model, it is important to describe each LULC in as much detail as possible, as this affects the accuracy of the model’s outcomes. Consequently, we reclassified some of the LULC types in LVR further, to better reflect their suitability for sustaining pollinators (Table S2). The *human-modified environment* or *built-up environment* was divided into 4 classes: *High built-up* (e.g., buildings), *Low built-up* ( e.g., highways and roads), *High urban vegetation* (e.g., urban trees and parks) and *Low urban vegetation* ( e.g., golf courses and campings). The threshold for height was set at 3 m following the criteria of the [Communauté Métropolitaine de Montréal (2017)](https://www.zotero.org/google-docs/?iEytDV). High and low vegetation have different nesting suitability, with lower vegetation being suitable mostly for cavity nesters, and high vegetation providing both ground and cavity nesting possibilities. Regarding impervious surfaces such as roads or buildings, flat surfaces are not suitable as nesting habitats, while higher buildings can provide some, albeit limited, nesting capacity.

The old fields and shrublands class was also reclassified according to the height of the vegetation into low (< 3 m) and high (≥ 3 m) vegetation. The rationale behind this is the same as that for the high and low vegetation in the built-up environment.

To reclassify the LULC map based on vegetation or building height, we used publicly available LiDAR images [(Ministère des Ressources Naturelles et des Forêts 2023)](https://www.zotero.org/google-docs/?9wHF7p), and Normalized Difference Vegetation Index (NDVI) data for the period May to August of 2014, obtained from the Google Earth Engine platform.

The *agricultural environment* was divided into three types. *Agricultural environment type 1* refers to crops that do not provide floral resources to pollinators, or that provide them to a lesser extent, or that are rotated with crops that do not provide floral resources (e.g., corn, soya, oat and wheat). *Agricultural environment type 2* includes crops that provide floral resources for pollinators (e.g., some vegetables and fruits). *Agricultural environment type 3* includes non-cultivated agricultural areas such as ditches, channels, and agricultural paths. Information of agricultural type was obtained from the C*artography of Land Occupation in the Lowlands of the Saint-Laurent*  (ECCC et MDDELCC, 2018), and complemented when necessary with the *Parcels and Reported Agricultural Productions* (PRAP) database (Financière Agricole du Québec 2023).

The *mixed-deciduous forest environment* was divided into two classes. The *mixed-deciduous forest edge* class refers to the first ten meters from the edge to the interior of the forest; the *Inner mixed-deciduous forest* class includes the entire interior part of the forest, from the 10-m edge to the center. The rationale behind this reclassification was that wild bees will find more feeding resources and nesting habitat at the edge of the forest than in the inner portion, where the canopy is denser. Information of forest type was obtained from the C*artography of Land Occupation in the Lowlands of the Saint-Laurent* (ECCC et MDDELCC, 2018).

Wetlands were separated by type: *wet meadows*, *swamps*, *forested peatlands*, *bogs and fens*, and *shallow water*, as each of these provide different amounts of floral and nesting resources. Information for wetland type was obtained from the C*artography of Land Occupation in the Lowlands of the Saint-Laurent* (ECCC et MDDELCC, 2018).

**S2. Limitations of the approach for identifying abandoned farmland**

As stated in the PRAP User's Guide [(La Financière Agricole du Québec 2023)](https://www.zotero.org/google-docs/?M1zqHv), this database is not complete or exhaustive. When this study was conducted, the database had not been fully updated for 2021. Nonetheless, the PRAP is considered the most complete source of information regarding cropland composition and extension in Quebec by researchers and practitioners from agricultural-related domains.

Until 2020, all crop fields that had been declared at least once as cultivated fields to *La Financière Agricole* were kept in the database, whether the fields were currently cultivated or not. Consequently, some crop fields may have been identified as abandoned in our analysis, when their actual status is, in fact, unknown.

In a limited number of cases, a field was described as cultivated in the PRAP database but the type of crop was not specified. The PRAP User’s Guide states that these are cases where information about the crop is either difficult to interpret, the crop was not declared that year, or it was defective (due to disease, abandonment or crop freezing). If no other information in the database indicated that the field was indeed cultivated, we considered it to be uncultivated. Although our approach could lead to false positives for abandoned lands, we believe that this type of error is not significant given the few cases where crop information was absent.

**S3. Limitations of the approach for identifying potentially degraded agricultural fields**

The use of NDVI trends as proxy for soil degradation has been questioned, as NDVI data can be influenced by numerous factors other than soil degradation: the soil type, spacing between vegetation rows, type of crop, rainfall and temperature variations, etc. [(Huang et al., 2014; Montandon & Small, 2008)](https://www.zotero.org/google-docs/?apvGR5). However, given that soil degradation negatively influences crop yield, we believe that the approach implemented here provides a good first approximation of soil degradation status. Within-field management to improve soil’s health will require more in-depth soil quality assessment.

It has been suggested that linear analysis on NDVI trends overlooks dynamic vegetation processes such as cyclic behaviors that might influence NDVI values over time [(Easdale et al., 2019)](https://www.zotero.org/google-docs/?G5P4mf). In our case study, the seasonal effect on NDVI has been eliminated by analyzing only satellite images in the growing and harvesting seasons.

NDVI trends may be also influenced by crop rotation in different years, given that different crop types have different intrinsic NDVI values. Crops in the study region are rotated in 3 to 5 year cycles, and we then assumed that crop rotation would not strongly influence the general trend in NDVI along the 22-year period considered in the analysis. We verified our assumption by visually inspecting the NDVI trends in random fields. If the methodology and/or maps presented in this research were to be used to, for example, develop management policy, final maps should be field validated.

**S4. Restoration scenarios**

***No restoration* scenario**: For the no restoration scenario, we used the LULC map of LVR, described in section S1.

***Reforestation in abandoned and degraded fields* scenario:** The crop fields that were identified as abandoned or degraded were reclassified as a mixed-deciduous forest class. Under this scenario, we did not differentiate between the inner portion and the edge of the forest and assigned the same categorical class to the abandoned and degraded fields and the edge of the mixed-deciduous forests.

***Flower strips on edges* scenario:** To simulate flower strips, we drew a 5-m wide polygon along the longest edge of crop fields in the LVR, using the PRAP database as a source for crop field polygons (year 2014) and QGIS [(QGIS Development Team 2024.)](https://www.zotero.org/google-docs/?A5zEf9) to draw the flower strip. To select the longest edge, we first separated each line composing a field’s polygon. Then we calculated the length of each line and selected the longest one. Careful visual validation showed that, in a small number of cases, the line identified as the longest edge of a field was not. This can probably be attributed to the fact that the vectorial shapefile of crop fields contains polygons that are highly irregular and in many cases contain a high number of nodes (and consequently, a high number of sides). Because of that, our procedure does not always select the longest edge of fields, nor always places strips perfectly on one side of the edge. We estimated that this was the case for around 18 % of the crop fields. Still, each crop field has a polygon representing a flower strip, and importantly, the total area covered by flower strips matches that of other scenarios, relevant aspects for comparison purposes. Given the small number of cases where this occurred, we disregarded this anomaly for our comparative and conceptual purposes. We removed flower strip polygons that were located on the edges of fields identified as abandoned or degraded. The flower strip polygons were converted to a raster format, creating a new LULC class. The raster map of flower strips was then merged over the *no restoration* scenario map (function *r.patch* from GRASS plugin in QGIS). This process reduced the resolution of the flower strips from 5 m to 30 m, but the total area of flower strips lost in this process represented only 0.003 % of the study area. We assumed that this loss of restored area was not relevant for purposes of our theoretical exercise.

***Mixed-strategy* scenario:** From a methodological point of view, the construction of this scenario followed the same procedure as the r*eforestation in abandoned and degraded fields* and the *flower strips on edges* scenarios. For the total area to be restored in this scenario to be equal to the r*eforestation in abandoned and degraded fields* and *flower strips on edges* scenarios (3% of the study area), we sub-selected which flower strips and which abandoned and degraded fields were to be restored. This selection procedure is explained in the main text.

***Maximal restoration* scenario**: To build this scenario we merged the r*eforestation in abandoned and degraded fields* and the *flower strips on edges* scenarios. We further removed flower strips located within 50 m of abandoned and degraded lands, since we assumed that flower strips are not likely to be placed near reforested patches. This scenario represents 5.8% of the study area restored: 3% as reforested patches in abandoned and degraded fields, and 2.8% as flower strips on field edges.

**S5. Pollination**

S5.1. InVest Crop Pollination model

The InVest pollination model, besides the LULC map, needs two input tables (the *guild table* and the *biophysical table*), which were parameterized as shown in Tables S1 and S2. Both tables were verified by six independent experts in pollination and wild bees of Quebec. Input parameters in the tables were then adjusted according to their suggestions.

The *guild table* (Table S1) defines the nesting preferences (as cavity or ground), season of activity, flying range, and relative abundance of bee species. We used the bee genera reported in [(Botzas-Coluni et al., 2021)](https://www.zotero.org/google-docs/?P7GhrZ) who sample bees in soya crop fields in the Montérégie, the administrative region of Quebec where LVR is located. Due to a lack of fine-scale data to parametrize the guild table, we opted to use relative values between 0 and 1, where a value of *zero* meant no nesting preference at all for a given nesting type, and a value of *one* meant absolute preference for that nesting type. We used cavity and soil nesting types alone for pollinators, for simplicity. Although this approach did not allow us to assess actual levels of pollination, it was suitable for comparing the increase in pollination provision among restoration scenarios.

The *biophysical table* (Table S2) defines the relative suitability of the different LULC types for providing nesting and feeding resources for pollinators. Each LULC type was assigned a value between 0 and 1 that reflects its relative capacity to provide nesting habitat and floral resources. We did not include in our model the differences in floral resource capacity between spring and summer as this was not relevant for the purposes of our work.

InVest's crop pollination model allows to calculate an index of yield attributable to wild pollinators. However, this yield outcome is still relative, and we believe it would add further information to our analysis, especially since the calculated yield relies on the model's bee abundance, and the relative pollinator dependence that we used to map supply and demand.

The input LULC maps used for the crop pollination InVest’s model included a 2-km buffer to avoid edge effects. Pollinators are central place foragers (and this is how the model is conceived), meaning that they go back to nests after foraging. The buffer is then needed to account for potential nesting sites right outside the border of the study area, but that could be the habitat of bees that forage within the study area borders.

**Table S1** Parameters for nesting preferences and season of activity in the InVest model. Parameters are relative values ranging from 0 to 1, with *zero* indicating no nesting preference for that type of nesting habitat or no activity in a given season, and *one* meaning absolute preference for that nesting habitat type or maximal activity in that season. We assumed that all species had the same relative abundance. The main references for the guild table parametrization were: [Agriculture and Agri-Food Canada (2014); Harmon-Threatt (2020); Natural Capital Project (2024); Packer et al. (2007); Zhao et al. (2019); and Zurbuchen et al. (2010)](https://www.zotero.org/google-docs/?IUhxXm)

| ***Bee Species (genera)*** | ***Cavity Nesting Preference*** | ***Soil Nesting Preference*** | ***Foraging activity (spring)*** | ***Foraging activity (summer)*** | ***Foraging Distance (m)*** |
| --- | --- | --- | --- | --- | --- |
| *Bumblebees* | *0, 25 0.25* | *1* | *0.5* | *1* | *1300* |
| *Augochlora* | *1* | *0* | *0.25* | *1* | *500* |
| *Andrea* | *0* | *1* | *1* | *0.5* | *500* |
| *Osmia* | *1* | *0* | *1* | *0.5* | *500* |
| *Ceratina* | *1* | *0* | *1* | *0.5* | *300* |
| *Halictus* | *0* | *1* | *0.5* | *1* | *300* |
| *Agapostemon* | *0* | *1* | *0.25* | *0.5* | *625* |
| *Chelostoma* | *1* | *0* | *0* | *1* | *1200* |
| *Melissodes* | *0* | *1* | *0* | *0.25* | *845* |
| *Colletes* | *0.25* | *1* | *0* | *1* | *740* |

**Table S2** The parameters in this table indicate the relative suitability of each LULC type for providing nesting habitat and floral resources for wild bees. A value of *zero* means no nesting capacity or no floral resources availability for all LULC types, while a value of *one* means highest nesting capacity or floral availability in that LULC class, relative to the other LULC types. The main references for the biophysical table parametrization were: [Groff et al. (2016); Koh et al. (2016); Schulp et al. (2014)](https://www.zotero.org/google-docs/?L3Ucbo). (*) *Agricultural environment type 1* refers to crops and cultures that potentially provide feeding resources for bees (e.g., berries, broccoli, tomatoes, etc.); (**) *Agricultural environment type 2* refers to crops that do not provide feeding resources for bees (e.g., wheat, sorghum, hay, etc.); (***) *Agricultural environment type 3* refers to small non-cultivated elements (e.g., small paths, drainage channels, etc.), according to the data source for the LULC map (ECCC et MDDELCC, 2018).

| ***LULC Class*** | ***Cavity Nesting Suitability*** | ***Soil Nesting Suitability*** | ***Floral Resources***  ***Availability*** |
| --- | --- | --- | --- |
| Low built-up impervious surfaces | *0* | *0.1* | *0* |
| High built-up impervious surfaces | *0.1* | *0* | *0* |
| Low urban vegetation | *0.1* | *0.25* | *0.5* |
| High urban vegetation | *0.5* | *0.5* | *0.5* |
| Low old fields and shrublands | *0.1* | *1* | *1* |
| High old fields and shrublands | *1* | *1* | *0.75* |
| Bare ground | *0* | *0* | *0* |
| Agricultural environment type 1(*) | *0* | *0.1* | *0.1* |
| Agricultural environment type 2 (**) | *0.1* | *0.25* | *0.75* |
| Agricultural environment type 3 (***) | *0.25* | *0.25* | *0.1* |
| Deep water | *0* | *0* | *0* |
| Mixed-deciduous forest (inner) | *0.75* | *0.5* | *0.1* |
| Mixed-deciduous forest (edge) | *0.75* | *0.5* | *0.75* |
| Coniferous forest | *0.25* | *0.25* | *0* |
| Wetland - Shallow water | *0* | *0* | *0* |
| Wetland - Marsh | *0* | *0* | *0.1* |
| Wetland - Swamp | *0.5* | *0* | *0* |
| Wetland - Wet meadow | *0* | *0* | *0.5* |
| Wetland - Forested peatland | *0.5* | *0* | *0.25* |
| Wetland - Fen | *0* | *0* | *0.25* |
| Flower strips | *0.5* | *1* | *1* |

S5.2. Pollination demand

To assess the demand for pollination of each cultivated crop field in LVR, we followed the methodology proposed by [Schulp et al. (2014)](https://www.zotero.org/google-docs/?ZNaYFv) and [Koh et al. (2016)](https://www.zotero.org/google-docs/?dydeZj). Pollination demand was measured at the field level as the area of the insect-pollination-dependent crop (i.e., the field area) weighted by the pollination-dependency level. Pollination dependency weights were drawn from [Klein et al. (2007)](https://www.zotero.org/google-docs/?2gWLn7): little dependency, 0.05; modest dependency, 0.25; great dependency, 0.65; essential dependency, 0.95. If more than one crop was cultivated in a field, we used the main crop (i.e., the one with the largest share within the field) as a reference for dependency, based on information obtained from [La Financière Agricole du Québec (2023)](https://www.zotero.org/google-docs/?yxlVY6).

**References**

Agriculture and Agri-Food Canada. (2014). Native pollinators and agriculture in Canada. Agriculture and Agri-Food Canada. https://www.publications.gc.ca/collections/collection_2014/aac-aafc/A59-12-2014-eng.pdf. Accessed 29 March 2024#

[Botzas-Coluni, J., Crockett, E. T. H., Rieb, J. T., & Bennett, E. M. (2021). Farmland heterogeneity is associated with gains in some ecosystem services but also potential trade-offs. *Agriculture, Ecosystems & Environment*, *322*, 107661. https://doi.org/10.1016/j.agee.2021.107661](https://www.zotero.org/google-docs/?vou8TY)

[Communauté Métropolitaine de Montréal. (2017). *Indice Canopée Métropolitain. Méthodologie*. https://observatoire.cmm.qc.ca/documents/geomatique/IndiceCanopee/2015/CMM_indiceCanopee_2015_methodologie.pdf](https://www.zotero.org/google-docs/?vou8TY)

[Easdale, M. H., Fariña, C., Hara, S., Pérez León, N., Umaña, F., Tittonell, P., & Bruzzone, O. (2019). Trend-cycles of vegetation dynamics as a tool for land degradation assessment and monitoring. *Ecological Indicators*, *107*, 105545. https://doi.org/10.1016/j.ecolind.2019.105545](https://www.zotero.org/google-docs/?vou8TY)

[ECCC et MDDELCC (2018). *Cartographie de l’occupation du sol des Basses-terres du Saint-Laurent, circa 2014.* Environnement et Changement climatique Canada et Ministère du Développement durable, de l’Environnement et de la Lutte contre les changements climatiques,, Plan d’action Saint-Laurent.](https://www.zotero.org/google-docs/?vou8TY) <http://data.ec.gc.ca/data/sites/systems/land-cover-mapping-of-the-st.-lawrence-lowlands/PASL_Occupation_sol_Rapport_methodologique.pdf>. Accessed 29 mars 2024.

[Groff, S. C., Loftin, C. S., Drummond, F., Bushmann, S., & McGill, B. (2016). Parameterization of the InVEST Crop Pollination Model to spatially predict abundance of wild blueberry (Vaccinium angustifolium Aiton) native bee pollinators in Maine, USA. *Environmental Modelling & Software*, *79*, 1–9. WorldCat.org.](https://www.zotero.org/google-docs/?vou8TY)

[Harmon-Threatt, A. (2020). Influence of Nesting Characteristics on Health of Wild Bee Communities. *Annual Review of Entomology*, *65*, 39–56. https://doi.org/10.1146/annurev-ento-011019-024955](https://www.zotero.org/google-docs/?vou8TY)

[Huang, J., Wang, H., Dai, Q., & Han, D. (2014). Analysis of NDVI Data for Crop Identification and Yield Estimation. *IEEE Journal of Selected Topics in Applied Earth Observations and Remote Sensing*, (11), 4374–4384. https://doi.org/10.1109/JSTARS.2014.2334332](https://www.zotero.org/google-docs/?vou8TY)

[Klein, A.-M., Vaissière, B. E., Cane, J. H., Steffan-Dewenter, I., Cunningham, S. A., Kremen, C., & Tscharntke, T. (2007). Importance of pollinators in changing landscapes for world crops. *Proceedings of the Royal Society B: Biological Sciences*, *274*(1608), 303–313. https://doi.org/10.1098/rspb.2006.3721](https://www.zotero.org/google-docs/?vou8TY)

[Koh, I., Lonsdorf, E. V., Williams, N. M., Brittain, C., Isaacs, R., Gibbs, J., & Ricketts, T. H. (2016). Modeling the status, trends, and impacts of wild bee abundance in the United States. *Proceedings of the National Academy of Sciences*, *113*(1), 140–145. https://doi.org/10.1073/pnas.1517685113](https://www.zotero.org/google-docs/?vou8TY)

[La Financière Agricole du Québec. (2023). *Base de données des parcelles et productions agricoles déclarées (BDPPAD). Guide de l’Utilisateur*.](https://www.zotero.org/google-docs/?vou8TY)

[Ministère des Ressources Naturelles et des Forêts. (2023). *LiDAR - Modèles numériques (terrain, canopée, pente)* [dataset]. https://www.donneesquebec.ca/recherche/fr/dataset/produits-derives-de-base-du-lidar](https://www.zotero.org/google-docs/?vou8TY)

[Montandon, L., & Small, E. (2008). The impact of soil reflectance on the quantification of the green vegetation fraction from NDVI. *Remote Sensing of Environment*, *112*(4), 1835–1845. https://doi.org/10.1016/j.rse.2007.09.007](https://www.zotero.org/google-docs/?vou8TY)

[Natural Capital Project (2024). *Crop Pollination InVest Model User Guide*. http://releases.naturalcapitalproject.org/invest-userguide/latest/en/croppollination.html](https://www.zotero.org/google-docs/?vou8TY)

[Natural Capital Project. (2024). *InVEST 3.14.1* [Computer software]. https://naturalcapitalproject.stanford.edu/software/invest](https://www.zotero.org/google-docs/?vou8TY)

[Packer, L., Genaro, J., & Sheffield, C. (2007). The Bee Genera of Eastern Canada. *Canadian Journal of Arthropod Identification*, *3*.](https://www.zotero.org/google-docs/?vou8TY)

[QGIS Development Team. (2024). *QGIS Geographic Information System*. QGIS Association. https://www.qgis.org](https://www.zotero.org/google-docs/?vou8TY)

[Schulp, C. J. E., Lautenbach, S., & Verburg, P. H. (2014). Quantifying and mapping ecosystem services: Demand and supply of pollination in the European Union. *Ecological Indicators*, *36*, 131–141. https://doi.org/10.1016/j.ecolind.2013.07.014](https://www.zotero.org/google-docs/?vou8TY)

[Zhao, C., Sander, H. A., & Hendrix, S. D. (2019). Wild bees and urban agriculture: Assessing pollinator supply and demand across urban landscapes. *Urban Ecosystems*, *22*(3), 455–470. https://doi.org/10.1007/s11252-019-0826-6](https://www.zotero.org/google-docs/?vou8TY)

[Zurbuchen, A., Landert, L., Klaiber, J., Müller, A., Hein, S., & Dorn, S. (2010). Maximum foraging ranges in solitary bees: Only few individuals have the capability to cover long foraging distances. *Biological Conservation*, *143*(3), 669–676. https://doi.org/10.1016/j.biocon.2009.12.003](https://www.zotero.org/google-docs/?vou8TY)
